# Trends in Notifiable Infectious Diseases in China: Implications for Surveillance and Population Health Policy

**DOI:** 10.1371/journal.pone.0031076

**Published:** 2012-02-16

**Authors:** Lei Zhang, David P. Wilson

**Affiliations:** The Kirby Institute for infection and immunity in society, Faculty of Medicine, University of New South Wales, Sydney, Australia; INSERM & Universite Pierre et Marie Curie, France

## Abstract

This study aimed to analyse trends in notifiable infectious diseases in China, in their historical context. Both English and Chinese literature was searched and diseases were categorised according to the type of disease or transmission route. Temporal trends of morbidity and mortality rates were calculated for eight major infectious diseases types. Strong government commitment to public health responses and improvements in quality of life has led to the eradication or containment of a wide range of infectious diseases in China. The overall infectious diseases burden experienced a dramatic drop during 1975–1995, but since then, it reverted and maintained a gradual upward trend to date. Most notifiable diseases are contained at a low endemic level; however, local small-scale outbreaks remain common. Tuberculosis, as a bacterial infection, has re-emerged since the 1990s and has become prevalent in the country. Sexually transmitted infections are in a rapid, exponential growth phase, spreading from core groups to the general population. Together human immunodeficiency virus (HIV), they account for 39% of all death cases due to infectious diseases in China in 2008. Zoonotic infections, such as severe acute respiratory syndrome (SARS), rabies and influenza, pose constant threats to Chinese residents and remain the most deadly disease type among the infected individuals. Therefore, second-generation surveillance of behavioural risks or vectors associated with pathogen transmission should be scaled up. It is necessary to implement public health interventions that target HIV and relevant coinfections, address transmission associated with highly mobile populations, and reduce the risk of cross-species transmission of zoonotic pathogens.

## Introduction

China has experienced a large decline in the spread and burden of infectious diseases since the early 1960s, associated with effective and large-scale public health interventions and large population-based vaccination programmes. China successfully eliminated 11 infectious diseases – including smallpox from the general Chinese population in early 1960s [1], 19 years before its global eradication – and another 10 infectious diseases, including poliomyelitis, have more recently been eliminated [2]. A further 13 diseases, including measles, are thought to be contained well at low endemic levels [3].

Surveillance systems for infectious diseases in China are mainly hospital based. The latest available statistics (from 2006) indicate that China has 18,703 county hospitals, 40,907 township hospitals and 201,562 medical clinics [4], [5]. Hospitals at the prefecture level or above are usually equipped with reference laboratories that are capable of carrying out molecular surveillance for cases. Together with laboratories from academic institutions, they form the front line of surveillance for outbreak detection and notification of infectious diseases in China. All hospitals and clinics are obliged to report both suspected and confirmed cases of notifiable infectious disease to their nominated county Centre for Disease Control (CDC). After recording the details of the reported cases (including their geographical location, demographicl information and infection status), county CDCs send the information to the country's central CDC, through the National Infectious Diseases Monitoring Information System Database, which was established in 2004 [6]. This reporting mechanism bypasses the previous stepwise hierarchical reporting framework, thus allowing information to flow directly from grassroots CDCs to China's central disease database. However, regional, provincial and national CDCs do not have equal access to all of the data in the database: their access rights are limited to their own administrative regions, and only national CDCs has the full access to all data [7]. In addition to their responsibility for verifying the disease information from their administrative regions, municipal and provincial CDCs are required to report to the appropriate level of the Bureaus of Health or Department of Health and to form networks with local research bodies, universities and other health organisations. At the top of the hierarchy, China's central CDC is the overseeing organisation with responsibility for assembling and analysing diagnostic data for all diseases and then presenting a final report to the Ministry of Health, which is the main public health policymaker in China. The central CDC is also the only legitimate office for disseminating infectious disease information to the public, but provincial and municipal CDCs are also authorised to publicise the information to people in their jurisdiction, under the supervision of the Ministry of Health [8]. In contrast to disease surveillance systems in Europe, the Chinese surveillance system uses a multilayer administrative mechanism that enables rapid and efficient upward flow of epidemic information.

China is a populous country of 1.3 billion people [9]. Over the last 30 years of economic reform in the country, there have been environmental, demographic, social and behavioural changes in the population. As China's ties with the rest of world become increasingly strong, infectious diseases in China no longer remain a domestic issue. A thorough review of infectious disease surveillance in China is timely and important for the country's disease prevention and control strategies. Currently, 39 infectious diseases are classified as notifiable in China. This study reviews trends in notifiable infectious diseases in China, in their historical context, discusses the current epidemiological state of these infections and their implications for disease surveillance and public health interventions. The review is structured by category of disease or transmission route.

## Methods

Currently, 39 infectious diseases are notifiable in China, classified as A, B or C according to their epidemic levels and potential population threats. Groups A and B (total 28 diseases) represent categories of diseases with high risk of outbreaks or that are likely to result in rapid spread once an outbreak occurs. Mortality and morbidity related to group A and B diseases are reported and published by the Chinese Ministry of Health on a monthly basis. Group C diseases are less infectious and, when outbreaks occur, are epidemiologically less severe. They are required to be reported only when outbreaks occur.

In this review, we searched published peer-reviewed research articles as well as online reports and grey literature from 1985 to 2010 relevant to disease surveillance in China in the following databases: PubMed, Chinese Scientific Journals Fulltext Database (CQVIP), China National Knowledge Infrastructure (CNKI) and Wanfang Data. Keywords used in the database search included [‘Chinese’ or ‘China’] and [‘Infectious diseases surveillance’ or ‘surveillance system’ or ‘infectious diseases monitoring’ or ‘infectious diseases information’ or the type of infectious disease or the name of individual infectious diseases]. We also searched governmental reports, reports of non-governmental organisations and other grey literature from online sources. We then collated data on notified cases and mortality related to these diseases, using the latest available information from the Ministry of Health [10]. Notifiable infectious disease data, including morbidity and mortality rates, was summarised by Chinese Ministry of Health and published annually in Chinese Health Yearbook and online accessible through CNKI database. Notably, this dataset does not contain data on SARS and influenza A(H5N1) virus infection. Hence we did not include them in our statistical analysis, but describe them in the text.

The selected diseases were categorised into eight major types according to their diseases characteristics and origins. The morbidity and mortality for each disease type were calculated as the sum of the corresponding rates of individual diseases. The total number of diagnosed and death cases were estimated by multiplying morbidity and mortality rates by the overall Chinese population in the study years. Case fatality rate was defined as the percentage of persons diagnosed with the disease who die as a result of the illness during the calendar year, and was estimated by dividing mortality rate by morbidity rate of the diseases. Further, for each individual disease, we calculate the disease-specific mortality rates among the Chinese population in five-year intervals (1999–2003 and 2004–2008). For each disease, the mean annual increase or decrease during 1999–2008, with 95% confidence intervals, is estimated, based on linear regression of logarithmic values of the number of annual death cases.

## Results and Discussion

### Trends in infectious diseases in China, 1999–2008

**Figure 1:**
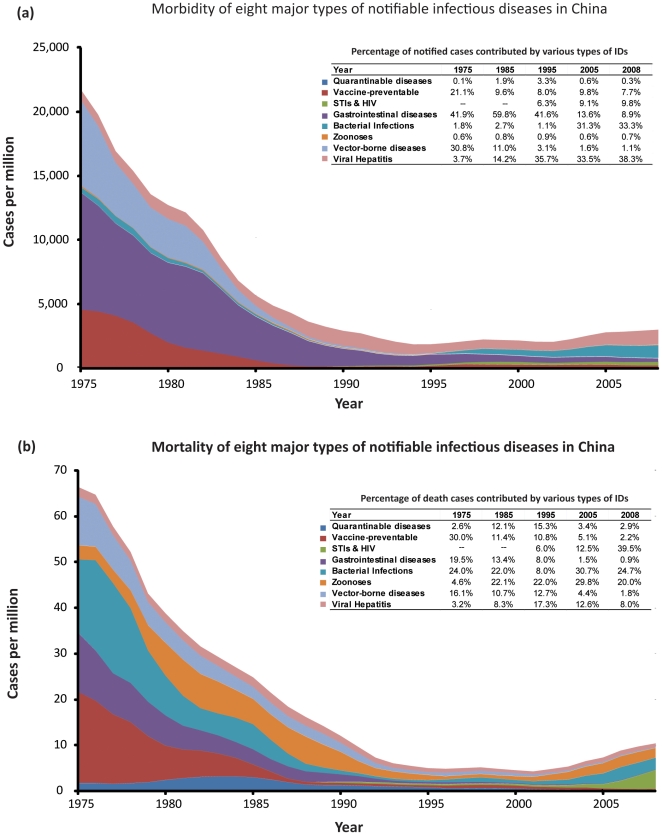
Temporal trend of morbidity and mortality rates, and their composition, of the eight major notifable infectious disease types in China.

Morbidity of notifiable infectious diseases in China, represented by estimated numbers of new cases, declined substantially (>90%) from 22,000 cases per million in 1975 to a nadir level of 1,800 cases per million in 1995 (Figure 1a). Since then, the rate of new infectious disease cases gradually reverted and maintained an upward trend. In 2008, the estimated rate of infectious diseases among the general Chinese population reached over 3000 cases per million population. The composition of diagnosed diseases cases also changed substantially, in 1975, the three most reported diseases were gastrointestinal diseases (41.9%), vector-borne diseases (30.8%) and vaccine-preventable diseases (21.1%), corresponding to a total of 93.8% of all diagnosed cases (Figure 1a). Additionally, these three types of diseases account for 35.5, 21.7 and 18.4 million reported cases respectively during the period 1975–1979 (Table 1). In contrast, in 1995, although gastrointestinal diseases remained the dominating disease type (41.6%), the proportion of all cases that were due to viral hepatitis quickly rose to 35.7%. Sexually transmitted diseases re-emerged, and together with HIV, consisted of a substantial 6.3% of all reported cases. In 2008, the three most frequently reported disease types included viral hepatitis (38.3%), bacterial infections (33.3%) and STIs and HIV (9.8%), which account for 5.4, 4.8 and 1.4 million diagnosed cases respectively during the period 2005–2008 (Table 1).

**Table 1:** Estimated number of diagnosed cases, death cases and case fatality rate of eight major infectious disease types in China in 5-year intervals.

Period	1975–1979	1980–1984	1985–1989	1990–1994	1995–1999	2000–2004	2005–2008
**Quarantinable diseases**							
Diagnosed cases	146,271	435,073	410,779	277,005	273,160	156,579	57,735
Death cases	8,190	14,694	10,636	5,879	3,757	2,192	1,382
Case fatality rate (%)	5.599%	3.377%	2.589%	2.122%	1.375%	1.400%	2.393%
[lower-upper limits]	[5.419–5.779%]	[3.295–3.458%]	[2.515–2.664%]	[2.040–2.204%]	[1.309–1.442%]	[1.311–1.489%]	[2.202–2.584%]
**Vaccine-preventable**							
Diagnosed cases	18,380,894	6,759,577	1,360,323	597,181	1,383,591	1,583,847	1,255,778
Death cases	72,545	28,885	7,085	3,130	3,987	3,684	1,409
Case fatality rate (%)	0.395%	0.427%	0.521%	0.524%	0.288%	0.233%	0.112%
[lower-upper limits]	[0.390–0.399%]	[0.420–0.435%]	[0.502–0.539%]	[0.496–0.552%]	[0.274–0.302%]	[0.221–0.244%]	[0.103–0.121%]
**STIs & HIV**							
Diagnosed cases	–	–	25,447	455,258	1,047,612	1,315,529	1,401,467
Death cases	–	–	108	1,568	1,763	2,838	12,533
Case fatality rate (%)	–	–	0.426%	0.344%	0.168%	0.216%	0.894%
[lower-upper limits]			[0.304–0.549%]	[0.318–0.370%]	[0.156–0.180%]	[0.204–0.228%]	[0.870–0.918%]
**Gastrointestinal diseases**							
Diagnosed cases	35,552,854	27,701,730	13,159,721	5,536,107	3,834,567	2,613,811	1,644,061
Death cases	46,153	23,698	13,909	5,257	2,230	1,298	593
Case fatality rate (%)	0.130%	0.086%	0.106%	0.095%	0.058%	0.050%	0.036%
[lower-upper limits]	[0.128–0.132%]	[0.084–0.087%]	[0.103–0.108%]	[0.091–0.099%]	[0.054–0.062%]	[0.046–0.054%]	[0.032–0.040%]
**Bacterial Infections**							
Diagnosed cases	2,258,134	1,047,059	503,691	202,619	1,476,435	3,363,049	4,820,142
Death cases	78,491	30,151	16,081	3,119	4,205	5,908	13,330
Case fatality rate (%)	3.476%	2.880%	3.193%	1.540%	0.285%	0.176%	0.277%
[lower-upper limits]	[3.439–3.512%]	[2.831–2.919%]	[3.118–3.267%]	[1.458–1.622%]	[0.272–0.298%]	[0.169–0.183%]	[0.269–0.284%]
**Zoonoses**							
Diagnosed cases	329,350	293,478	315,863	141,292	73,625	54,194	97,626
Death cases	17,354	35,607	29,295	12,528	4,496	8,161	11,420
Case fatality rate (%)	5.269%	12.133%	9.275%	8.867%	6.107%	15.060%	11.698%
[lower-upper limits]	[5.152–5.386%]	[11.952–12.313%]	[9.120–9.429%]	[8.640–9.094%]	[5.842–6.371%]	[14.599–15.520%]	[11.390–12.007%]
**Vector-borne diseases**							
Diagnosed cases	21,731,070	10,533,882	1,690,125	537,490	266,424	228,970	216,301
Death cases	37,200	19,283	13,262	7,081	4,232	2,863	1,375
Case fatality rate (%)	0.171%	0.183%	0.785%	1.317%	1.589%	1.250%	0.636%
[lower-upper limits]	[0.169–0.174%]	[0.179–0.187%]	[0.764–0.805%]	[1.271–1.364%]	[1.516–1.661%]	[1.181–1.320%]	[0.584–0.687%]
**Viral Hepatitis**							
Diagnosed cases	4,359,763	4,507,954	5,599,627	5,592,120	3,897,266	4,400,935	5,388,845
Death cases	9,158	9,950	10,463	6,656	4,616	4,572	4,657
Case fatality rate (%)	0.210%	0.221%	0.187%	0.119%	0.118%	0.104%	0.086%
[lower-upper limits]	[0.203–0.217%]	[0.214–0.227%]	[0.181–0.192%]	[0.115–0.123%]	[0.113–0.124%]	[0.099–0.108%]	[0.083–0.090]

Rapid declines in infectious diseases mortality and its similar saddle pattern were also observed in the past 35 years. The overall mortality rate in China decreased from 66 cases per million in 1975 to 5 cases per million in 1995, then it gradually reverted to 10 cases per million in 2008 (Figure 1b). Vaccine-preventable diseases, bacterial infections and gastrointestinal diseases were the greatest causes of death, accounting for 30.0%, 24.0% and 19.5% of reported infectious diseases death cases among Chinese population in 1975 (Figure 1b). However, the rank of composition shifted to zoonoses (22%), viral hepatitis (17.3%) and quarantinable diseases (15.3%) in 1995. Since then, the proportion of deaths caused by STIs and HIV, and bacterial infections rapidly increased. By 2008, STIs and HIV (39.5%) has become the mostly deadly infectious disease, followed by bacterial infections (24.7%) and zoonoses (20.0%). During the period 2005–2008, these three types of diseases have led to approximately 12,500, 13,300 and 11,400 deaths in China, respectively (Table 1).

Case fatality rate measures the percentage of deaths among people who contracted a disease. During 1975–2008, the disease type with the highest fatality rate is zoonoses, consistently causing 5–15% of deaths among the infected population. Following that, quarantinable diseases killed 1.4–5.6% of its infected population during the same period. In comparison, fatality rates in vaccine-preventable (0.112% during 2005–2008), gastrointestinal diseases (0.036%), bacterial infections (0.277%) and viral hepatitis (0.086%) show clear decreasing trends in China, corresponding to 3.5, 3.6, 12.4 and 2.4 folders reduction in comparison with the period 1975–1979, respectively. However, fatality of STIs and HIV increased to 0.894% during 2005–2008, doubling the level in 1985–1989 (Table 1).

### Quarantinable diseases

Plague, cholera and epidemic haemorrhagic fever (which is caused by hantaviruses and mainly transmitted by rodents) have had diverse impacts on the Chinese population at different stages of recent history. During 1944 to 1949, there were 179 notified outbreaks of plague across China, which resulted in an estimated 2.4 million deaths. From 1950 to 1954, the mean number of annual notifications of plague infection was 1,373 cases per year [11], [12]. This was then substantially reduced to 20 cases per year over the following 30 to 40 years. Since 1990, the number of annual reported plague cases has increased to approximately 52, which is equivalent to 0.004 per 100,000 population per year [11], [12]. There is no observed trend in the mean annual mortality due to plague during 1999–2008 (Table 2).

**Table 2:** Mortality rates and temporal trends in notifiable infectious diseases^a^ in China, 1999–2008.

Notifiable infectious diseases	Mean Annual Mortality rate per 100,000 population		Mean annual increase or decrease during 1999 to 2008	
	1999–2003	2004–2008	Percentage (%)	95% confidence interval
**Quarantinable diseases**				
Plague	0.0100	0.0036	−22	−57 to +41
Cholera	0.0081	0.0002	−48*	−70 to −12
Epidemic haemorrhagic fever	0.0235	0.0159	−12*	−20 to −3
**Vaccine-preventable diseases**				
Poliomyelitis	0.0001^b^	0.0001^b^	0	0 to 0
Measles	0.0101	0.0058	−6	−20 to +10
Pertussis	0.0081	0.0003	−44*	−59 to −23
Diphtheria	0.0080	0.0080	0	−60 to +153
Tetanus	0.0323	0.0174	−12*	−2 to −8
**Gastrointestinal diseases**				
Bacillary and amoebic dysentery	0.0155	0.0087	−10*	−18 to −1
Typhoid fever/paratyphoid fever	0.0086	0.0029	−22*	−39 to −2
**Vector-borne diseases**				
Epidemic/endemic typhus	0.0080	0.0001^†^	−54	−87 to +61
Japanese encephalitis (scrub typhus)	0.0254	0.0196	−6	−14 to +3
Visceral leishmaniasis (kala-azar)	0.0075	0.0035	−5	−69 to +20
Malaria	0.0087	0.0040	−14	−26 to +1
Dengue fever	0.0100	0.0051	−42*	−80 to +66
**Zoonotic infections**				
Leptospirosis	0.0112	0.0040	−20*	−32 to −6
Brucellosis	0.0100	0.0035	−24	−64 to +63
Anthrax	0.0083	0.0025	−31*	−100 to −1
Rabies	0.0753	0.2153	26*	+14 to +37
**Bacterial infections**				
Meningococcal meningitis	0.0099	0.0126	3	−2 to +8
Scarlet fever	0.0080	0.0034	−23	−66 to +74
Tuberculosis	0.0580	0.2244	28*	+14 to +43
**Sexually transmitted infections**				
HIV infection	0.0285	0.2185	44*	30 to 58
Gonorrhoea	0.0100	0.0002	−49*	−63 to −30
Syphilis	0.0083	0.0059	−4	−19 to +15
**Viral hepatitis**				
All	0.0697	0.0897	5*	2 to 8
Hepatitis A	0.0059	0.0025	−21*	−32 to −8
Hepatitis B	0.0473	0.0642	9*	1 to 20
Hepatitis C	0.0027	0.0076	30*	16 to 46
Hepatitis E	0.0019	0.0031	9	−3 to +24

AIDS: acquired immunodeficiency syndrome; HIV: human immunodeficiency virus.

*Denotes a statistically significant (p<0.05) mean annual increase or decrease of disease-specific mortality rates during 1999 to 2008.

a Group A and B notifiable diseases.

b The detection limit of the mortality rate is 0.0001 per 100,000 population.

**Figure 2:**
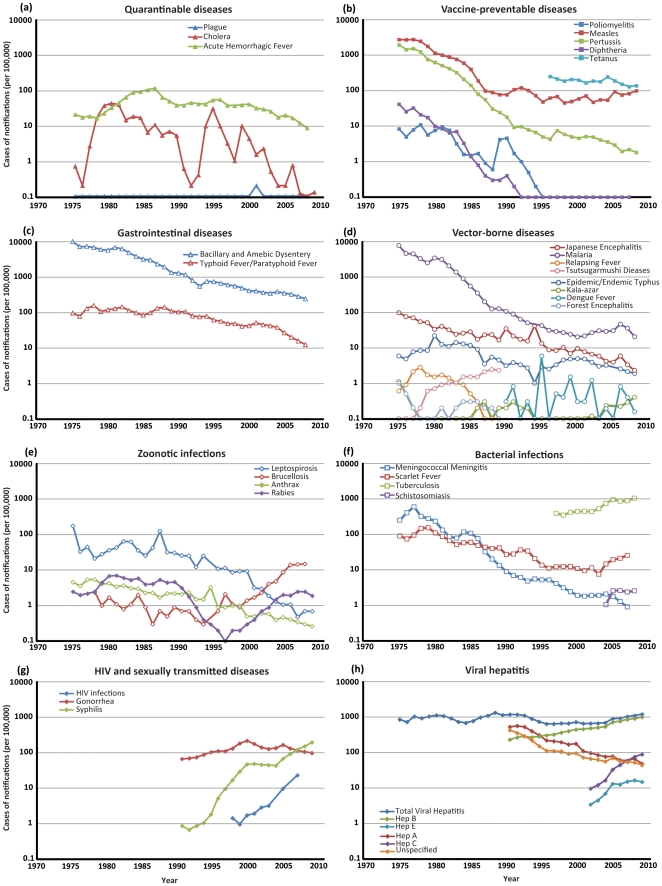
Incidence of notified cases of infectious diseases^a^ in China by category of disease, 1975–2008.

Cholera has caused two major outbreaks in China in recent decades: the first started in 1973, leading to a large peak in the number of cases in 1980, with an annual notification rate of four cases per 100,000 population (Figure 2a). The rate then gradually subsided but there was a second major outbreak in the early 1990s. During 2004 to 2008, cholera cases were reported at relatively low incidence levels: the disease is well controlled, with a mean annual decline in mortality due to the disease of 48% (95% CI, 12–70%, Table 2).

Despite a 13-fold decrease in the epidemic haemorrhagic fever notification rate since 1987 and continuous decline in the disease-specific mortality rate, China is still the country most severely affected by this disease, with 90% of the global cases of haemorrhagic fever with renal syndrome (HFRS) occurring in China [13], [14]. HFRS remains an important public health issue in China, as an estimated 20,000–50,000 new cases are reported each year [15]. It is important to establish effective strategies to reduce the incidence of HFRS. The mean annual decline in mortality due to this disease is 12% (95% CI, 3–20%, Table 2).

Persistent public health campaigns have been effective in reducing the number of rodents, which has been largely responsible for the decreasing trend in plague and epidemic haemorrhagic fever notifications. Improved hygiene and sanitary conditions have also led to the decline in incidence and mortality due to cholera.

Overall, these quarantinable diseases persist at low endemic levels, but still pose potential threats.

### Vaccine-preventable diseases

In the early 1960s, a nationwide vaccination programme was implemented to eradicate smallpox, measles, poliomyelitis, tuberculosis (TB), pertussis, diphtheria and tetanus. Measles vaccine (one-dose schedule) was introduced in the country in 1965, and from 1978 it has been administered routinely to all infants through the country's expanded programme on immunisation [16]. Measles mortality and morbidity has declined continuously and substantially since 1978. During 1995 to 2007, the incidence was less than one per 100,000 population (Figure 2b), with fewer than 250 deaths due to measles reported annually [17]. However, limited small-scale outbreaks continue to occur among susceptible children in rural areas with low routine immunisation coverage and among susceptible children of ‘floating immigrants’ (the relatively large population of internal Chinese immigrants who leave their rural hometown and move to urban areas to seek better employment opportunities) [18]. In addition, although a two-dose measles vaccination schedule has been recommended in China since 1986, the second dose is widely perceived as a non-mandatory booster. Coverage of the second dose is not reported, but is thought to be low [19].

A global effort to eradicate polio began in 1988 [20]), wild-type poliovirus was eradicated in China in 1994 [21], [22]; extensive surveillance for acute flaccid paralysis is thought to have strongly contributed to polio eradication in the country [23].

Vaccination against diphtheria, pertussis and tetanus was also made mandatory for all infants under the country's expanded immunisation programme since 1960s. In 2006, vaccination coverage of the vaccine against the three diseases reached 99.0%, comparable with polio vaccination (99.0%) and measles vaccination (98.6%) [24]. Since 2006, the total annual incidence of reported cases of diphtheria, pertussis and tetanus decreased to below 0.5 cases per 100,000 population (Figure 2b). Widespread vaccination against these diseases is one of the important contributors to improvements in child health in China: infant mortality rate dropped from 20% in 1950 to 1.7% in 2006 [24]. Mandatory vaccination of children throughout the past decades has been demonstrated to be successful in containing the spread of these diseases in China. It is estimated that polio vaccination alone has saved a total of 1,128,000 children in China from disabling disease and suffering [24]. Mortality due to pertussis and tetanus experienced significant annual decays at rates of 44% (95% CI, 23–59%) and 12% (95 CI%, 30–65%) (Table 2). Trends in TB are discussed in the section on bacterial infections.

### Gastrointestinal diseases

The number of reported cases of bacillary and amoebic dysentery has declined rapidly since 1975 in China (Figure 2c). An initial large decline occurred in the mid-1970s, largely due to substantial improvements in sanitary conditions and quality of drinking water. However, approximately 84 million diarrhoeal episodes were still reported in China each year at the end of the 1990s: 25% of the episodes were in children under five years of age [25]. *Shigella* bacilli and *Entamoeba histolytica* have been found to be the main aetiological organisms [25]. A live oral *Shigella* vaccine was then developed in 1997 in China, which provided 60–70% protection against *S. flexneri* serotype 2a and *S. sonnei* infection [26]. Between 1999 and 2008, reported deaths due to dysentery decreased by a mean of 10% (95 CI%, 1–18%) per year (Table 2).

The number of notified cases of typhoid and paratyphoid fevers is very low in China, with an annual rate of 1.2 notifications per 100,000 population in 2008 (Figure 2c). The number of reported deaths due to these infections has decreased at a mean rate of 22% (95% CI, 2–39%) per year (Table 2). However, these infections remain endemic in some rural areas of southern China, where there is a lack of adequate sewage disposal and safe water supplies [27]. A large, localised outbreak occurred during 1996 to 1998 in Xing-An county in the southern province of Guangxi, which resulted in an annual incidence rate of 39 to 103 cases per 100,000 population [28]. Subsequently 61,030 doses of Vi polysaccharide vaccine were administered in 1999 and the epidemic quickly subsided. No further typhoid fever outbreaks were reported in China.

### Vector-borne diseases and parasitic diseases

National public health campaigns since 1949 in China effectively improved hygiene and sanitary conditions, which substantially reduced most of vector-borne infectious diseases. In 1955, a total of 5,970,000 malaria cases were reported nationally, accounting for 68% of the total number of reported infectious disease cases and an incidence of 1,028 per 100,000 population. Malaria disproportionately affected some regions, with incidence rates as high as 10,360 per 100,000 population in Hainan province [29]. In 1960 and 1970, there were major outbreaks of the disease, leading to a national incidence of 1,554 and 2,961, respectively, per 100,000 population [29]. It took considerable time for the incidence to fall: the number of cases did not decrease to 1955 levels until 1982, but it has since continued to decrease. By 1998, the rate of notifications decreased to 31.3 per 100,000 population, corresponding to more than a 99% reduction and accounting for only 1.3% of the total number of reported cases of notifiable conditions [29]. Data for 2004 to 2008 indicate that the mean annual mortality rate due to malaria was reduced to 0.004 per 100,000 population (Table 2), corresponding to only 52 reported deaths per year countrywide. Malaria no longer represents a major threat to the Chinese population. The ongoing government-mobilised mass movement for vector control is probably an effective method for eliminating mosquitoes and their habitats and consequently reducing the disease burden due to malaria in the country. The universal healthcare system provided an effective base for the implementation of countrywide interventions [6].

As a result of the national public health campaigns to eliminate infectious disease vectors, forest encephalitis (also named Russian spring-summer encephalitis), tick-borne relapsing fever, tsutsugamushi disease (also known as scrub typhus) and epidemic typhus were reduced to very low levels by the late 1980s and were then no longer notifiable. The number of visceral leishmaniasis (also known as kala-azar), Japanese encephalitis, malaria and dengue fever epidemics has also decreased, with few cases per year (Figure 2d). However, there are signs that climate change may have led to local outbreaks of some these vector-borne diseases in the past decade in China [30].

### Zoonotic infections

Rabies is one of the zoonotic diseases on the rise in China. During a 58-year period between 1950 and 2007, a total of 117,530 human rabies cases were reported, corresponding to an incidence rate of 9.1 cases per 100,000 population. Three major epidemics were observed [31], [32]: the first occurred in the mid-1950s when the annual number of reported cases reached a peak of about 2,000. The epidemic subsided in the 1960s and then started to surge again in the early 1970s. After reaching a sustained peak from 1982 until the end of the decade, at a level of 5,000 to 6,000 cases per year [33], in 1996 the number of cases was at its lowest, with 159 reported cases [33]. As the disease in humans is closely associated with transmission of rabies virus by infected dogs, in the past epidemics, strict policies for vaccination of owned dogs and elimination of stray dogs were put in place, resulting in effective control of the epidemic. However, since 2000, due to a new wave of rapid expansion of the pet dog population in urban China, there has been a third outbreak, in which the number of cases has increased rapidly: in 2007, the number of notified cases climbed to 3,250 [34], [35]. The rabies-related mortality rate increased during 1999 to 2008 by a mean of 26% (95% CI, 14–37%) per year (Table 2). This should serve as a call for action to prevent the further spread of rabies in China.

Severe acute respiratory syndrome (SARS), caused by a coronavirus that was transmitted to humans through close contact with civet cats, spread from Guangdong, China, quickly leading to a worldwide epidemic in 2003. China reported 5,327 (66%) of the 8,071 SARS cases globally and 349 SARS-related deaths during the epidemic, which lasted for approximately a year [36].

The first human case of highly pathogenic avian influenza A (H5N1) virus infection was reported in Hong Kong in 1997. By 2009, mainland China had 88 avian influenza outbreaks among birds in 23 provinces and a total of 38 human cases and 25 deaths had been reported [37]. In 2010, the epidemic had spread to 15 countries and resulted in 296 deaths, out of 500 cases [38]. In comparison, the 2009 influenza A(H1N1) pandemic caused more than 154,000 human cases and 842 deaths in China alone [39], despite the virus being much less virulent than influenza A(H5N1) virus. China, with concentrated human activity in its fast-growing major cities that may lead to effective transmission of human-to-human transmission of H1N1, is very vulnerable to the transmission of respiratory infectious diseases of zoonotic origin. During the mid-to-late stages of the SARS outbreak and, more apparently throughout the course of the 2009 influenza pandemic, the Chinese Government responded swiftly with openness and transparency to control both diseases and prevent further episodes. In both cases, the epidemics caused widespread social panic. Instead of the Ministry of Health, the State Councils – representing the highest administrative authority of the Chinese Government – assumed direct leadership in combating the epidemics.

Case notifications for leptospirosis, anthrax and brucellosis have remained at low levels since 2000. There was an increase in the number of notified case of brucellosis from 0.17 per 100,000 population in 2000 to 1.50 per 100,000 population in 2007, but this was probably due to improved surveillance and is believed not to reflect an increase in incidence [40]. The annual number of notified cases of leptospriosis and anthrax dropped below 0.01 per 100,000 population in 2008. The mortality rate due to brucellosis remains very low and stable, whereas the rates due to leptospriosis and anthrax have dropped significantly, by 20% and 31%, respectively, during 1999 to 2008 (Table 2).

### Bacterial infections

China has the second largest TB epidemic in the world (after India) [41]. In 2008, some 4,500,000 patients were living with pulmonary TB in China, corresponding to an incidence of 111 per 100,000 population, and resulting in 130,000 deaths from TB in the same year [42]. Since 2005, an estimated 20,000 additional TB reactivation cases have arisen per annum in China due to human immunodeficiency (HIV) coinfection [42].

The TB epidemic has spread unevenly in China: in 2005, China reported a national TB prevalence of 0.2% [41], whereas the southern province of Guangxi, which borders Vietnam, reported a much higher prevalence of 0.65% [43]. In 1991, in adopting DOTS – the internationally recommended TB control strategy – China launched a 10-year infectious and endemic disease control project in an effort to curb its expanding TB epidemic in 13 of its 31 provinces [44], [45]. It is estimated that the number of Chinese people infected with TB between 1990 and 2000 decreased by 30% and that 300,000 TB deaths were averted. With a success rate greater than 90% [45], DOTS has led to the avoidance of an estimated 1.5 million TB cases [44], [45]. However, since 2000, multidrug-resistant (MDR) TB and extensively drug-resistant (XDR) TB have emerged as a severe public health issue in the country. In 2004, China reported approximately 140,000 MDR TB cases, accounting for about one third of the estimated global burden of MDR TB [46]. In some provinces, the proportion of MDR TB among newly notified TB cases and previously treated cases was found to exceed 10% and 30%, respectively [47]. The latest study indicates that the prevalence of MDR TB among TB patients is as high as 19.4%, and 14.9% of MDR TB cases were XDR during 2007 to 2009 [48]. The mortality due to TB increased at a rate of 28% (95% CI, 14–43%) per year.

Hydroelectric projects that led to the formation of a large number of dams in China, as well as climate change, have substantially increased the risk of schistosomiasis occurring and its spread to non-endemic areas of the country, leading to small-scale outbreaks and increases in rates of case notifications near the affected areas [49], [50]. The incidence of schistosomiasis has increased from 0.10 cases per 100,000 population in 2004 to 0.26 per 100,000 population in 2008 (Figure 2f).

### HIV and other sexually transmitted infections

Since the founding of China in 1949, laws have been enacted to make commercial sex illegal. Brothels were closed down and sex workers were sent to camps to be ‘re-educated’. Special institutions for treatment of sexually transmitted infections (STIs), at the time mostly syphilis, gonorrhoea and Chlamydia, were established for the training of medical personnel, who were then sent to areas previously associated with large sex industries to treat STIs at beginning of 1950s. During 1950s, a universal healthcare system was established that provides treatment for STI patients free of charge. Designated laboratories for STIs were built for diagnosis and research [51]. More importantly, population-based health education campaigns about STIs were carried out, and detection and treatment of STIs were portrayed as patriotic actions [52]. As a result, the number of cases of notifiable STIs decreased from 10 million in 1950 to reportedly eliminated from 1964 [51], [53], [54]. However, notifiable STIs have re-emerged, leading to a fast-spreading epidemic in the country. In 1987, the incidence for STIs was 6.64 per 100,000 population and in 1996, the rate increased to 34.6 per 100,000 population, which corresponded to 1.8 million new STI cases [52]. The annual growth rate of the number of STI cases in China exceeds 20% [52]. The rise of STI incidence is mainly due to unprotected sexual acts and other high-risk behaviours [55], [56].

Since early 1980s, underground prostitution has again become rampant in major cities in China [57]. The number of Chinese men, increased purchasing power of urban residents and trends away from traditional Chinese family values of fidelity all led to an increased number of male clients for commercial sexual services [58]. In addition, lack of sexual health knowledge among commercial sex workers and their inability to effectively negotiate condom use during sexual acts substantially increased the risk of STI transmission [57], [59]. The previously hidden population of men who have sex men (MSM) is becoming more overt due to increasing acceptance in society. High-risk sexual behaviours, including a low percentage of condom use, in this population is also contributing to the fast transmission of STIs [60]. For example, in 1998, the incidence of syphilis in China was 0.17 per 100,000 population: it increased 20-fold to 4.31 cases per 100,000 population in 2002 and further to 15.88 cases per 100,000 population in 2007 [10], [51] (Figure 2g). In particular, MSM, injecting drug users (IDUs) and female sex workers have the highest syphilis prevalence in China, of 10–20% [61], [62], 5.4% [63] and 7–12% [64], respectively.

The first case of acquired immunodeficiency syndrome (AIDS) in China was reported in 1985, but a local HIV epidemic was not detected until 1989 when a cluster of infections was diagnosed among IDUs in Yunnan province [65]. Before 2000, China had fewer than 20,000 reported HIV/AIDS cases, but in 2007, the number rose to 200,000. The latest estimates are that 700,000 people were living with HIV/AIDS in China at the end of 2007, with 70,000 new infections added every year since then [66] and 20,000–30,000 annual AIDS-related deaths [67]. Although the HIV/AIDS epidemic first began and predominated among IDUs, it rapidly spread to other population groups through both heterosexual and homosexual transmission. The latest figures demonstrate that heterosexual transmission has accounted for approximately 38–50% of new HIV infections since 2005 [68], which may level at or exceed the percentage (30–49%) caused by transmission due to the sharing of injection equipment among IDUs [69], [70]. Notably, HIV infection has become more prevalent among MSM, as male homosexual activities account for approximately 10% of new infections [71]. AIDS mortality rate increased from 0.10 in 1999 to 1.01 cases per million in 2005, then to 4.58 cases per million in 2008, corresponding to an annual growth rate of 44% (95% CI, 30–58%) the rate is expected to increase sharply if the epidemic remains uncontrolled and there is no large procurement of antiretroviral drugs. Cumulatively, over 9000 infants were infected through mother-to-child transmission by 2005 and it was estimated that about 500 million Chinese were carriers of *Mycobacterium tuberculosis* in 2003 [72]: coinfection of HIV and *M. tuberculosis* could therefore have a large epidemiological impact, in the absence of appropriate public health interventions.

### Viral hepatitis

Viral hepatitis is prevalent in China, but the prevalence differs according to the type of virus. Before the 1990s, hepatitis A virus (HAV) was the most prevalent. In the early 1990s, the annual number of reported HAV diagnoses was more than 50 per 100,000 population. This dropped by 90% to 5 per 100,000 population in 2005 to 2006 [73] (Figure 2h). The mean annual death rate due to HAV decreased at a rate of 21% (95% CI, 8–32%) during 1999 to 2008 (Table 2). The decrease is probably a result of mass vaccination efforts in introducing the HAV vaccine (H2 strain) in the 1990s and improvement of hygiene and sanitary conditions that broke the cycle of food and water contamination [73]. According to 2008 data [74], HAV persists at low, endemic levels (Figure 2h). In comparison, infections of hepatitis B virus (HBV), which is transmitted mainly through body fluid from mother to child, and hepatitis C virus (HCV), transmitted predominantly by intravenous injections or blood transfusion, are becoming alarmingly widespread in China. The annual number of new HBV cases increased rapidly from 20 per 100,000 population in 1990 and reached 100 per 100,000 population in 2008, despite an increasing vaccination coverage of newborns from 30% in 1992 to 93.4% in 2005 [75]. HCV infections followed a similar trend and in 2007, the annual notification rate was about 8.8 per 100,000 population. The 2007 HCV and HBV prevalence among the general population was 0.5% and 6%, respectively [74]. Mortality due to HBV and HCV increased at annual rates 9% (95% CI, 1–20%) and 30% (16–46%) respectively.

In contrast to HAV infection, which always causes acute hepatitis but never develops into a chronic condition, HCV and HBV infections often cause chronic hepatitis and may develop into cirrhosis and hepatocellular carcinoma [76]. Coinfection of HBV or HCV with other pathogens causing chronic infections, such as HIV, results in substantial increases in the disease burden on individuals, accelerates disease progression and complicates treatment [77], [78]. Since HBV and HCV can both be transmitted through exchange of body fluids, coinfection of these hepatitis viruses with HIV is common in at-risk groups in China. Approximately 67–71% of the 700,000 registered IDUs in China are infected with HCV [79], [80], [81] and 17–26% of them are infected with HBV [79], [82]. The prevalence of HIV/HCV coinfection is 6.45% in IDUs [80]. Among blood donors, the prevalence of HIV/HCV coinfection is 5.8–6.5% [80], [83], whereas the figure for HIV/HBV coinfection is 3.7% [84]. In comparison, coinfection of HIV and HCV among the general population remains unknown as there is currently no reports on its prevalence. However, coinfection is expected to be increasingly noticeable as the HIV epidemic transforms into a generalised epidemic in China [6].

### Implications of disease surveillance and interventions in China

Overall, this study investigated the temporal trends of major types of notifiable infectious diseases in China. Our analysis indicated that while the morbidity and mortality of most infectious diseases reduced substantially during 1975–1995, there is an increasing trend of re-emergence of previously prevalent diseases and emergence of new infectious diseases since 1995, in particular, STIs and HIV, viral hepatitis and zoonoses diseases. These diseases accounted for the majority of deaths associated with infectious diseases in China since 2005. Consistent with this, analysis of case fatality rates indicated a higher percentage of deaths among zoonotically infected people (5–15%) and an elevated proportion (0.894%) among STIs and HIV-infected individuals. Our study has important implications for the surveillance and control of infectious diseases in China. First, the persistent small outbreaks of particular infectious diseases across the country indicate that any decrease in prevention efforts will probably trigger re-emergence of the diseases. Surveillance activities are therefore essential to inform and prioritise public health responses. Although efficient and well-developed disease surveillance systems have been implemented in many urban areas, hygiene conditions, health services and monitoring of patterns of spread and disease burden are largely lacking in rural China [6].

Second, the rapid rise in the number of notified cases of STIs, especially HIV infection, and viral hepatitis in China is associated with growth of the sex industry, increasingly frequent risky sexual behaviours and an increasing number of sexual partners in the general Chinese population. The newly implemented internet-based National Infectious Diseases Monitoring Information System Database not only provides a platform for integrating epidemiological data on HIV infection and other STIs, but also initiated second-generation behavioural surveillance of the at-risk populations [70]. Our analysis indicates that both epidemiological and behavioural surveillance of HIV infection and other STIs is essential to understand and forecast the trends in these epidemics. Extending surveillance efforts into population groups that were previously considered to be less at risk may further improve the quality and reliability of surveillance data.

Third, coinfections, especially those involving HIV, are likely to become major public health issues in the future. We demonstrate that TB/HIV, HBV/HIV and HCV/HIV coinfections have become increasingly prevalent in China and the trend is likely to continue in the absence of strong public health interventions. Thus, inclusion of testing for TB and viral hepatitis coinfections as part of HIV voluntary counselling and testing could be useful among at-risk populations. Scaling up of free-of-charge antiretroviral therapy should also include treatment for these coinfections.

Fourth, surveillance of zoonotic infections becomes increasingly important due to the interactions between humans and animals in China. China has established an efficient surveillance system for human infectious diseases [6], [85]: a parallel national centralised system for surveillance of disease in animals may be useful for reducing the risk of animal-to-human transmission.

One of the potential limitations in our investigation may be poor data quality in the passive notification-based surveillance system. It is widely perceived that underreporting exists in the Chinese infectious diseases surveillance system, but little is known about the extent of underreporting as systematic evaluations have never been conducted [6]. This issue requires further consideration in the future. An overview of prevalence studies would be useful for comparison with numbers of notified cases in order to estimate numbers of undiagnosed cases. A comprehensive evaluation would also involve assessments of the efficiency and effectiveness of the surveillance system, both quantitative and qualitative. Understanding changes in testing rates in various population groups will also be useful for interpreting trends in notification data. But such investigations are beyond the scope of this review.

### Future challenges

Infectious disease surveillance and interventions in China face several major challenges. The country's major economic reform in 1979 has created a large economic disparity between rural and urban areas, resulting in a large population of floating immigrants (also known as ‘peasant workers’). This accounts for 10% of the total Chinese population in 2005 [86]. The vast majority of these immigrants are from rural environments but work in urban areas, away from their families, and hence regularly travel between the two sites. In addition to the natural population growth of major cities, the inflow of internal immigrants substantially increases the population size and density in urban areas. These immigrants often live in overcrowded residential areas with compromised hygiene conditions. Female immigrants often become targets of those seeking to recruit them for commercial sex work [57], [87]. These floating immigrants are often more disadvantageous than the local residents. They tend to be less educated compared with others in urban areas who have permanent resident status and less likely to seek medical treatment when sick due to financial constraints or lack of health insurance [6]. Not surprisingly, the population of floating immigrants has been found to be more vulnerable to infectious diseases, especially STIs, HIV and viral hepatitis [6]. Additionally, their high-risk behaviours and mobility promotes transmission of infectious disease agents and creates a major challenge for disease detection and control. Given the characteristics, behaviours and the increasing population size of floating immigrants, it becomes apparent that the capacity of current Chinese healthcare system cannot meet the needs of both the surveillance activities and health problems of these migrant workers. The infectious disease surveillance system needs to be tailored for its high mobility, and a dramatic expansion of the private health sector is required to address the health needs of this population.

Transmission of zoonotic infectious agents relies on close interaction between infected animals and humans. As a result of the increasing social trend of keeping house pet dogs, it is estimated that the number of pet dogs in China reached 80–200 million in 2004 [88]. However, only 30% of dog owners register their animals with government authorities and only 2–8% of dogs are vaccinated against rabies [31], [89] – coverage of greater than 70% is needed to sufficiently control rabies, as determined by the World Health Organization (WHO) [90]. The high rabies prevalence (6.4%) among dogs and the expanding number of pet dogs pose a continual and increasing threat of transmission of rabies virus from dogs to humans in China [89]. In addition, the consumption of game meat, especially in southern China, is one of the important sources of zoonotic diseases [91]. Markets in Guangdong provinces sell live poultry, fish, reptiles and mammals, including dogs and civet cats, for food [92]: the housing of these live animals, often in packed conditions, together with human activities, makes such markets the ideal hub for cross-species transmission of zoonotic agents [91]. Since there is no systematic surveillance of these live markets, it is difficult to estimate the number of such markets and the amount of game meat consumed by residents in southern China. The SARS epidemic in 2003 demonstrates that transmission of pathogens originally associated with animals can cause worldwide outbreaks in human populations. The current absence of both epidemiological and behavioural data, including their frequency of contacts and means of animal handling, on animal infectious diseases is a strong barrier to effective surveillance and control.

Environmental changes facilitate the emergence and transmission of bacterial infections. The formation of a large number of dams in China, as a result of the increasing implementation of hydroelectric projects, have substantially increased the risk of schistosomiasis emergence and its spread to non-endemic areas of the country. Further, the dense population conditions in urban China and the high mobility of its floating migrants substantially promote the rapid transmission of tuberculosis [93]. Strengthening infectious diseases surveillance for bacterial infections among these specific affected populations should be a priority for the Chinese government.
